# A Case of Tubo-Ovarian Abscess in a 15-Year-Old Female After Appendectomy Complicated by Peritonitis

**DOI:** 10.7759/cureus.20052

**Published:** 2021-11-30

**Authors:** Maryellen Campbell, Ahmed A Noor, Martin Castaneda

**Affiliations:** 1 Obstetrics and Gynecology, Florida Atlantic University, Schmidt College of Medicine, Boca Raton, USA; 2 Obstetrics and Gynecology, Bethesda Hospital East, Boynton Beach, USA

**Keywords:** pyosalpinx, diagnostic laparoscopy, peritonitis, appendectomy, stump appendix, appendicitis, pelvic inflammatory disease, tubo-ovarian mass

## Abstract

Pelvic inflammatory disease (PID) and tubo-ovarian abscess (TOA) are serious diagnoses to consider in reproductive-age women presenting with abdominal or pelvic pain. Management can be medical or surgical depending on severity. This case report outlines the unique presentation of TOA in a 15-year-old female presenting with acute abdominal pain with a recent past surgical history of appendectomy. A discussion of the approach to similar presentations and the importance of maintaining a broad differential diagnosis follows.

## Introduction

A tubo-ovarian abscess (TOA) is a feared complication of pelvic inflammatory disease (PID) involving abscess formation in the uterus, fallopian tube, and ovaries. Infection can develop due to an ascending vaginal infection or from a gastrointestinal infection as not all cases of TOA are associated with PID. Between 15-35% of women treated for proven PID will also be diagnosed with TOA [1]. Most women with TOA are of reproductive age. The estimated prevalence of lifetime self-reported PID is 4.4% in sexually active women of reproductive age [2]. TOAs tend to be polymicrobial with commonly implicated organisms including *E. coli*, *B. fragilis*, *Prevotella*, and *Peptostreptococcus*. The differential diagnosis of a TOA includes ovarian torsion, ectopic pregnancy, appendicitis, gastroenteritis, constipation, and urinary tract infection (UTI).

Management of TOA may be medical, surgical, or both and should be aggressive as the risk of delayed treatment can predispose to rupture or sepsis. Medical management usually involves intravenous (IV) antibiotics with an empiric regimen selected on the basis of most likely organisms. Patients are usually placed on two or three agents to allow for adequate coverage. Unfortunately, intravenous antibiotic therapy can have limited efficacy with large abscesses due to poor penetration into the target tissue. Progression to surgical treatment is necessary after poor response to antibiotics or in the setting of hemodynamic instability at presentation. Minimally invasive approaches are emphasized depending on pre-operative imaging findings. Open techniques can be considered for complicated pathology or large abscesses.

## Case presentation

A 15-year-old nulliparous female with noncontributory past medical history presented to the emergency department (ED) at our institution with acute onset abdominal pain that worsened over two days. She rated her pain as 5/10 and denied nausea, vomiting, diarrhea, fever, and chills. A recent appendectomy was performed five months ago which was complicated by postoperative peritonitis. At that time, she developed a transaminitis likely secondary to an inflammatory process resulting from her acute suppurative appendicitis. Her maximum alanine aminotransferase (ALT) was 149 and her maximum aspartate aminotransferase (AST) was 184 with an elevated C-reactive protein (CRP) of 4.2. She required IV metronidazole and piperacillin/tazobactam followed by an outpatient course of cefdinir and metronidazole for ten days. Her total duration of antibiotic therapy post-surgery was 14 days. She recovered after the appendectomy and did not have any further post-operative issues until her current presentation to the ED for abdominal pain. Her only previous surgery was the appendectomy five months prior. She does not take any medications and has no known drug allergies. She denies smoking, alcohol use, and drug use. She also denies sexual activity. Her family medical history is noncontributory.

Her vitals in the ED were stable with a temperature of 99.6 F. She was alert and oriented without acute distress. Her cardiac and lung exams were within normal limits. Upon abdominal exam, she was tender to palpation with positive guarding and rebound. She had active bowel sounds and no costovertebral angle tenderness. There was no edema of the extremities or calf tenderness. Her distal peripheral pulses were 2+ bilaterally.

**Figure 1:**
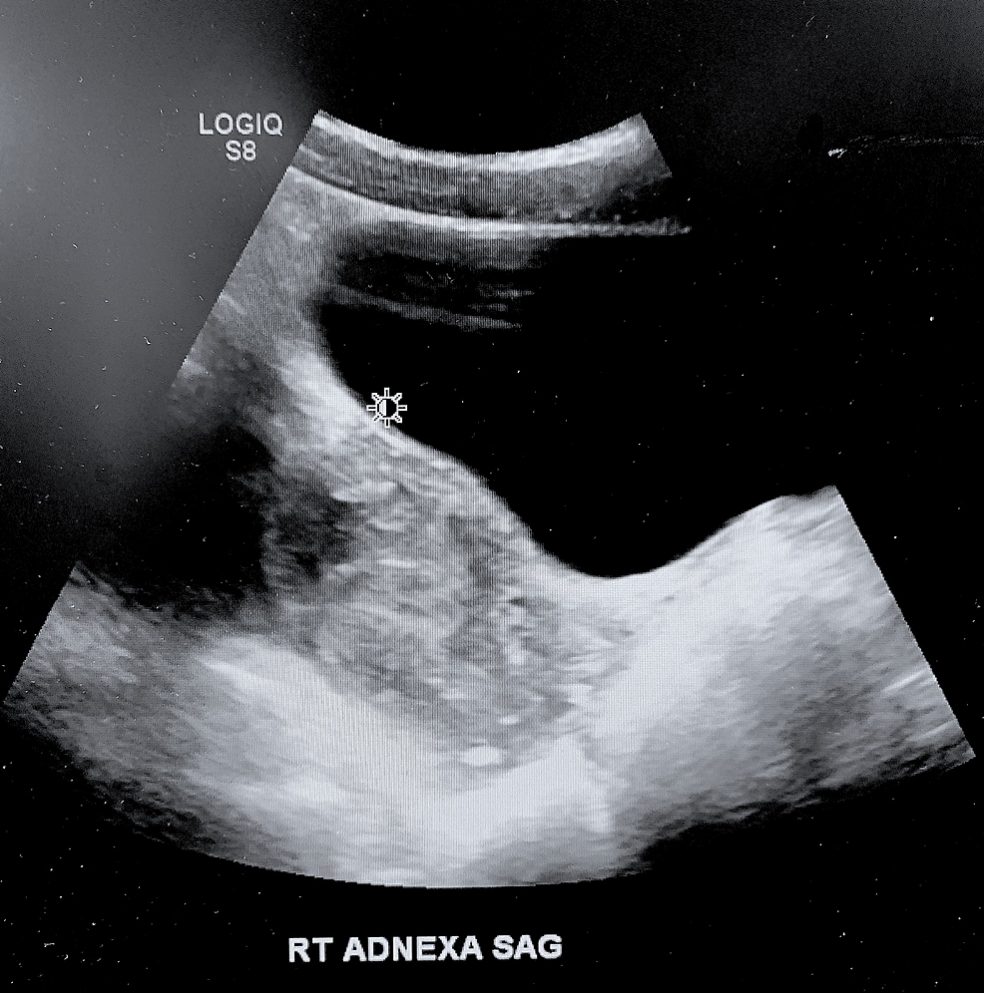
Pelvic ultrasound showing heterogenous mass adjacent to right ovary.

Her complete blood count (CBC) and complete metabolic panel (CMP) drawn in the ED were within normal limits. Urinalysis revealed 1+ leukocytes, 1+ protein, and all other findings within normal limits. Ultrasound in the ED showed a complex right annexation lesion adjacent to the right ovary measuring 7.1 cm x 4.3 cm x 4.3 cm with heterogenous echogenicity and a central anechoic fluid collection (Figure 1). It was believed this may represent an organizing clot or abscess formation. Abdominal X-ray revealed a non-obstructive bowel gas pattern.

The patient was taken to the operating room (OR) with a pre-operative diagnosis of a hemorrhagic ovarian cyst. A diagnostic laparoscopy was performed with drainage of the right pyosalpinx and subsequent removal of the stump appendix by general surgery. The stump appendix was evaluated by pathology and found to be a portion of benign appendix without evidence of neoplasia or an active inflammatory process. The pyosalpinx was comprised of necrotic material with acute inflammatory debris and no evidence of neoplasia. Other findings observed during the surgery included purulent fluid in the cul-de-sac, a normal right ovary, a normal left fallopian tube, and a normal left ovary. The post-operative diagnosis was amended to right pyosalpinx and stump appendix.

Post-operatively the patient did well. Her pain was controlled, and her Jackson-Pratt drain had minimal serosanguinous output. Antibiotics were continued, including doxycycline, metronidazole, and cefoxitin. Her diet was advanced to regular. She voided after the foley catheter was removed and passed flatus. She was discharged to home and followed up in the outpatient setting.

At the first follow-up appointment, the patient was again asked about her sexual history. She denied previous sexual activity. A cervical culture procured at this time was negative for gonorrhea and chlamydia.

## Discussion

Acute abdominal pain in the female pediatric population requires consideration of a broad range of differential diagnoses involving many organs including but not limited to ovaries, uterus, stomach, small and large bowel, appendix, bladder, ureters, kidneys, pancreas, liver, gallbladder, spleen, lungs, and neurologic or psychiatric causes. TOA is an urgent issue that cannot be missed and requires prompt initiation of intravenous antibiotic therapy due to both short-term complications such as peritoneal spread and systemic inflammatory response progressing to sepsis and long-term complications like chronic pelvic pain and future infertility [3]. Empiric, broad-spectrum antibiotic therapy includes antibiotics active against gram-positive, gram-negative, and anaerobic microbes due to the often polymicrobial nature of TOA [4]. Approximately one-third of patients will require surgical intervention [4]. This case of TOA in a virginal 15-year-old female status post appendectomy and peritonitis highlights the complexity of identifying a source of infection for TOA in adolescents. Fewer than 15 cases of TOA in non-sexually active females have been reported in the literature.

Our patient denied any history of sexual activity when interviewed in the inpatient and outpatient settings. However, it is reasonable to consider that an adolescent may not be forthcoming about sexual history especially in the presence of a parent for fear of punitive familial repercussions. Therefore, it is possible that the source of infection for this TOA was a sexually transmitted infection (STI) leading to PID. Furthermore, a cervical culture was not taken from the patient until her outpatient follow-up appointment approximately one week after discharge from the hospital. It is possible the antibiotics she was prescribed eliminated any evidence of STI that may have previously been present. The 2015 Centers for Disease Control and Prevention guidelines recommend parenteral inpatient treatment with a second- or third-generation cephalosporin, doxycycline, and metronidazole for severe pelvic inflammatory disease requiring inpatient admission [3].

Cases of TOA from hematogenous spread of gastrointestinal pathology and subsequent seeding of ovarian tissues have been described in the literature but are exceedingly rare [4]. Given the timeline of acute appendicitis five months before the presentation of symptoms attributed to the TOA in our patient, it is possible but very unlikely that the TOA resulted from seeding of the peritoneum after the appendicitis and resulting peritonitis. Most abscesses that develop after laparoscopic appendectomy develop on average nine days after surgery [5]. A previously reported case identified a 17-year-old non-sexually active female that developed TOA after laparoscopic appendectomy for a perforated appendix [6]. Her postoperative course was complicated by abdominal pain, fever, and leukocytosis [6]. It is extremely unlikely that the appendiceal and peritoneal inflammation in our patient five months prior is responsible for the development of this TOA.

The prognosis of TOA is variable ranging from no complications to recurring PID to development of pelvic pain or infertility [3]. The risk of infertility is dependent on the severity of the infection and the number of lifetime episodes of PID [7]. Close follow-up with a gynecologist is important in the setting of TOA. Ultrasound screening may be necessary after antibiotic treatment is complete to ensure resolution [4].

## Conclusions

TOA is a potentially dangerous complication of PID with many other conditions on the differential in a young female patient. This case of a 15-year-old female with abdominal pain and a recent surgical history illustrates key points in the diagnosis and management of TOA. It is generally advised to maintain a broad differential diagnosis especially in the setting of recent surgery or unclear social history. As TOA can be a potentially fatal condition, it is important to maintain a high index of suspicion in key demographic groups especially given the myriad ways in which the condition can emerge, including ascending vaginal flora, STI, and gastrointestinal seeding.
